# Depiction of Resuscitation on Medical Dramas: Proposed Effect on Patient Expectations

**DOI:** 10.7759/cureus.14419

**Published:** 2021-04-11

**Authors:** Cindy C Bitter, Neej Patel, Leslie Hinyard

**Affiliations:** 1 Surgery, Division of Emergency Medicine, Saint Louis University School of Medicine, St. Louis, USA; 2 Medicine, College of Human Medicine, Michigan State University, East Lansing, USA; 3 Health and Clinical Outcomes Research, Saint Louis University School of Medicine, St. Louis, USA

**Keywords:** patient education, mass media, narrative medicine, cultivation theory, social cognitive theory, self-efficacy, cardiac arrest outcome

## Abstract

The public has unrealistic views regarding the success of cardiopulmonary resuscitation, and one potential source of misinformation is medical dramas. Prior research has shown that depictions of resuscitation on television are skewed towards younger patients with acute injuries, while most cardiac arrests occur in older patients as a result of medical comorbidities. Additionally, the success rate of televised resuscitations on older shows has vastly exceeded good outcomes in the real world. We sought to understand resuscitation outcomes on current medical dramas and to review the literature for evidence that media affects patient decision-making. We reviewed medical dramas to evaluate the demographics of cardiac arrest victims and the success rate of resuscitations and compared the results to outcomes for real-world patients. Medical dramas continue to focus on trauma as the main cause of cardiac arrest and portray favorable outcomes more frequently than should be expected. Patients who believe the overly optimistic prognoses portrayed on television may be more likely to desire aggressive medical care in the face of serious illness. Healthcare workers should anticipate the need to counter misinformation when discussing patient goals of care and end-of-life planning.

## Introduction

It is well established that the public has unrealistic views of patient outcomes after cardiopulmonary resuscitation (CPR), with multiple studies showing laypeople believe the survival rate exceeds 40-50% [1-4]. One potential source of misinformation is media, particularly televised medical dramas. It has been hypothesized that unrealistic depictions of patient outcomes on medical dramas may affect patients’ expectations of success and cultivate unrealistic expectations [5-6]. Overly optimistic portrayals of CPR outcomes on television may contribute to patients' desire to pursue aggressive measures in the face of life-threatening illness. When elderly patients are given accurate information about prognosis after cardiac arrest, they are less likely to choose resuscitation [7]. 

Evidence that media influences behavior comes from multiple aspects of health such as impaired driving, substance abuse, and cancer screening [8-10]. A systematic review found 19 studies on the impact of medical dramas on health behavior [11]. The majority of studies displayed the mixed effects of medical dramas on health knowledge, perceptions and behaviors: 32% had beneficial effects, and 11% had a negative influence. Health narratives, such as television storylines, may have an effect on viewers that is out of proportion to objective risks and benefits [12].

*Chicago Med *and *Code Black* premiered in 2015 and joined the long-running *Grey’s Anatomy* as medical dramas on major network television in the United States. *Chicago Med *was created by veteran producer Dick Wolf, known for including storylines “ripped from the headlines” on his shows. *Code Black* took its name from a critically acclaimed documentary based on a busy public hospital in Los Angeles., featuring the tagline “Good Medicine. Great Drama.” Millions of viewers in the US watched these shows during their runs in prime time, and streaming platforms have subsequently increased the number of viewers. In contrast to television series with more fantastical elements such as *House* and *Scrubs*, *Code Black* and *Chicago Med* are presented to viewers as being more “realistic” and, thus, viewers might interpret outcomes within the show as reflecting real-world scenarios.

Given that the general population’s understanding and expectations of CPR outcomes are likely influenced by television narratives, this paper reviews outcomes of CPR on medical dramas on major broadcast networks in the 2015-16 and 2016-17 seasons and compares television outcomes to current real-world data, as well as providing a review on the impact of television habits on the viewer-patient’s health behavior.

## Materials and methods

*Chicago Med* (41 episodes, NBC), *Code Black *(34 episodes, CBS), and *Grey’s Anatomy *(48 episodes, ABC) ran during the 2015-16 and 2016-17 seasons. We watched all episodes and noted demographic information regarding depictions of CPR with an estimate of patient age group, etiology of arrest, resuscitation attempts, and outcomes. Special circumstances such as advanced directives and end-of-life discussions were noted when depicted. Information was entered on a Google Sheets spreadsheet.

Resuscitation attempts were defined as chest compressions or defibrillation of unconscious patients, artificial respirations, or other actions taken to revive a patient who was referred to as “arresting” or “coding”. All resuscitation attempts were included, regardless of whether they occurred in the pre-hospital setting, the emergency department, the operating room, or during hospitalization. Estimation of age group was made either objectively by dialogue or subjective assessment individuals as pediatric (less than 18 years), adult (between 18 and 64 years), and elder (65 years and above). The etiology of the arrest was coded as medical vs. traumatic. Outcomes were coded as alive with favorable neurologic outcome or moderate disability (FNO), alive with severe disability, persistent vegetative state (PVS) or brain death, death during resuscitation, delayed in-hospital death, and outcome unknown. Any patients seen to be awake and talking after CPR were coded as survival with FNO. Patients who were shown or described as suffering brain damage or having died during hospitalization were coded as such. Patients who survived the initial resuscitation attempt but did not meet the above criteria were coded as outcome unknown. The groups were chosen to correspond to dichotomized scores on the Cerebral Performance Category tool (CPC), which is widely used to track outcomes in resuscitation research [13]. A CPC score of 1 indicates no or mild impairments, a patient is alert and able to work, 2 indicates moderate disability but the patient is conscious and independent in activities of daily living, 3 indicates severe disability with a patient who is reliant on others for care, 4 indicates a patient in a coma or persistent vegetative state, and 5 indicates brain death. This is frequently separated into patients with FNO (CPC scores 1-2) and unfavorable neurologic outcome (CPC scores 3-5).

Real-world comparison data were selected by reviewing the literature for current demographics, etiologies of cardiac arrest, and outcome data. Outcomes after cardiac arrest are dependent on multiple factors: etiology of the cardiac arrest, patient age and comorbidities [14-15], the circumstances of the cardiac arrest [16-17], and characteristics of the emergency medical service and hospital system that care for the patient [18-19]. Given this variability, registry data that report outcomes for large cohorts of cardiac arrest victims were used as real-world comparators [20-25].

## Results

A total of 106 resuscitation attempts were included, with at least one resuscitation attempt portrayed in 64 of the 123 episodes. Of the 106 resuscitations, 18 (17%) were pediatric cases, 82 (77.4%) were adults, and 6 (5.8%) were elders. Medical causes of cardiac arrest accounted for 47 (44.3%) instances of CPR and traumatic cases accounted for 59 (55.7%).

On medical dramas, 14.9% of medical instances of cardiac arrest occurred in pediatric characters, 76.6% in adults, and 8.5% in elders. The Cardiac Arrest Registry to Enhance Survival (CARES) database includes 23 statewide registries and select communities from 18 additional states, capturing 115 million Americans. McNally et al. reviewed the demographics of non-traumatic cardiac arrests using the CARES database [25]. In their study, pediatric cases accounted for 1.9% of cardiac arrest victims, adults age 18-64 for 47%, and elderly for 51.1% (Table 1).

**Table 1:** Age of Medical Cardiac Arrest

	Pediatric	Adult	Elder
Chicago Med	4	11	1
Code Black	0	20	3
Grey’s Anatomy	3	5	0
TV total	14.9%	76.6%	8.5%
McNally et al	1.9%	47.0%	51.1%

**Figure 1:**
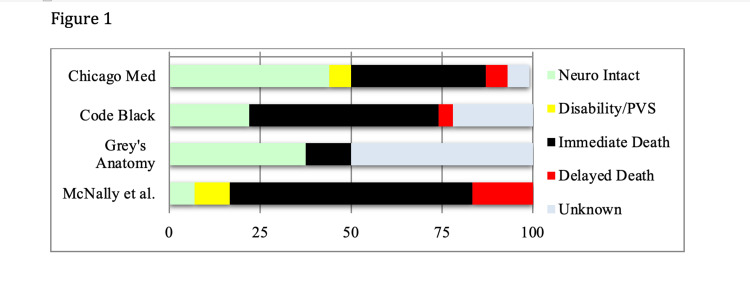
Outcomes After Medical Cardiac Arrest

Survival with FNO occurred in 32% of medical resuscitations on medical dramas, survival with disability or PVS occurred in less than 2%, death during resuscitation occurred in 38%, and delayed death occurred in 4% of medical resuscitations. Outcomes were unknown for 21% of medical resuscitations. Using the CARES database, McNally et. al. found FNO for 6.9%, survival to hospital discharge in 9.6% (corresponding to survival with disability of 2.7%). Survival to hospital admission was 26.3%, which corresponds to death during resuscitation of 73.7%. The majority of cardiac arrest patients did not achieve return of spontaneous circulation [25]. Characters on medical dramas were 4.6 times more likely to survive with FNO (Figure 1).

On medical dramas, 55.7% of cardiac arrests were due to trauma. In two European studies of cardiac arrest registries, trauma accounted for less than 3% of all cardiac arrests [21, 24]. Pediatric cases accounted for 20.3% of traumatic cardiac arrests on medical dramas, adults for 76.2%, and elders for 3.4%. Although victims of traumatic cardiac arrest tend to be younger than victims of medical etiologies, a German registry of traumatic cardiac arrest had 10.6% pediatric cases, 49.4% adult cases, and 40.0% cases in elders [24] (Table 2).

**Table 2:** Age of Traumatic Cardiac Arrest

	Pediatric	Adult	Elder
Chicago Med	4	13	0
Code Black	5	29	1
Grey’s Anatomy	3	3	1
TV total	23.3%	76.2%	3.4%
Gräsner et al.	10.6%	49.4%	40.0%

**Figure 2:**
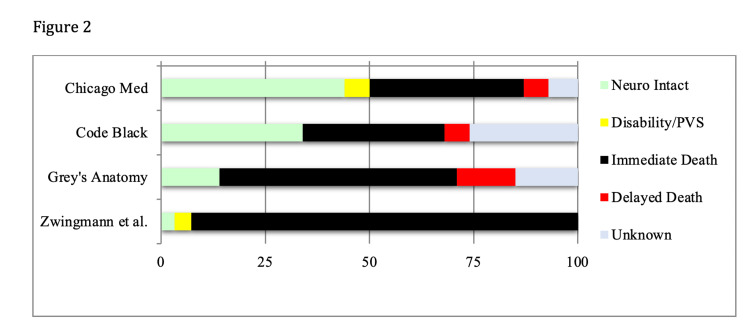
Outcomes After Traumatic Cardiac Arrest

On medical dramas, 29% of characters with traumatic cardiac arrest had survival with FVO, 2% had survival with disability, death occurred for 50%, and outcomes were unknown for 19%. In their systematic review, Zwingmann et al. found an overall survival rate of 7.2% in traumatic cardiac arrests, with FNO in 3.2% and survival with disability in 4%; they did not separate deaths during resuscitation and delayed in-hospital mortality [23]. Results are similar in more recent registry studies from Sweden [21], the US [22], and Germany [24]. A study from Japan found no survivors among pediatric victims of cardiac arrest due to motor vehicle collisions if patients did not have a pulse on arrival to the hospital [20]. Characters on medical dramas were nine times more likely to survive with good neurologic outcomes than real patients (Figure 2).

End-of-life discussions and patients with advanced directives are relatively rare on US medical dramas. Each show portrayed a resuscitation where physicians violated a known Do Not Resuscitate order. *Chicago Med *showed several instances of characters who survived the initial resuscitation attempt but had care withdrawn later in the episode; two of these characters were mentioned as being organ donors.

## Discussion

In this study, characters with medical causes of cardiac arrest were 4.6 times more likely to survive with good neurologic outcomes than patients in the real world while characters with traumatic cardiac arrest were nine times more likely. Medical dramas continue to misrepresent the demographics, etiologies, and outcomes of cardiac arrest.

Diem et al. examined depictions of CPR on television, using television dramas and a reality show from the US, and found that characters suffering cardiac arrest were significantly younger than real-world victims, more likely to have trauma as a cause of the cardiac arrest, and more likely to have survival with FNO than real-world victims [5]. They suggested that a focus on “miracle” cases accounted for the unrealistic outcomes. Portanova et al. reviewed US television dramas from 2010-2011 and found similar results [26]. Both studies commented on the paucity of advanced directives and end-of-life discussions. A more recent study confirmed that survival rates of cardiac arrest victims remain above 60% in a large number of television medical dramas, and that the quality of CPR technique is generally poor [27]. Our study confirms that the depiction of resuscitation after cardiac arrest continues to be skewed.

Medical dramas may influence health beliefs in the population in several direct and indirect ways. Several theoretical models have been proposed to explain this relationship. Cultivation theory postulates that repeated media exposure normalizes rare events, altering viewers’ perception of the likelihood of these outcomes. In one example, increased exposure to action movies was associated with taking more risks in traffic [8]. Social cognitive theory proposes that second-hand exposure to consequences of health behaviors portrayed in media affects viewers' behavior, particularly if the viewer does not have direct experience with the condition. College students exposed to reality television programs that glorified substance abuse were more likely to engage in illegal drug use [9]. Self-efficacy refers to the individual’s perceived ability to achieve health goals by changing their behavior. Kim and Hmielowski manipulated the voiceover in a television clip to highlight the ease or frustrations of obtaining screening for cervical cancer, where female college undergraduates who were randomized to the high self-efficacy voiceover were more likely to express intent to undergo screening [10]. Entertainment education theory suggests that viewers gain health knowledge by engagement with the storyline and identification with characters, and viewers emulate behaviors portrayed as beneficial to health [11].

Fewer studies have found that media can affect health beliefs and behavior directly relating to cardiac arrest. Nava et. al. found patients who viewed educational television programming had more accurate estimates of survival after CPR than those who got their information from medical dramas, newspapers, or the internet [2]. On the negative side, high school students who watch medical dramas overestimate survival rates after CPR [28], and regular viewers of medical dramas scored lower on tests of proper CPR technique [29]. Although embedding reliable public health messaging in US television has been proposed, Brusse et al. suggest viewers may resent and reject messages they perceive as biased [30]. The representation of medical procedures and outcomes, specifically in regards to resuscitation, is therefore important to consider in the context of medical dramas. It is important to further explore the specific influence of resuscitation outcomes on health beliefs.

Limitations

There are limitations to this study. We reviewed three medical shows however, *Code Black* is now canceled, and new medical dramas are now being broadcast. While the shows reviewed by this study are widely enjoyed by viewers, they do not represent all medical dramas. Furthermore, these results may not apply to reality programs, cable shows, daytime television, or programming from other countries.

Ultimately, there is limited data on how/if patients use info from fictional television shows to make medical decisions, especially in the demographics where these decisions are most likely to be required.

## Conclusions

Medical dramas in the United States portray CPR outcomes that are not consistent with real-world outcomes. Favorable outcomes are overrepresented in medical and traumatic resuscitations. The unrealistic depiction of CPR outcomes on medical dramas may contribute to public misinformation about resuscitation outcomes and reluctance to adopt advance directives through a variety of mechanisms, but data to support this hypothesis is limited.
